# Ca^2+^ channel blockade reduces cocaine’s vasoconstriction and neurotoxicity in the prefrontal cortex

**DOI:** 10.1038/s41398-021-01573-7

**Published:** 2021-09-06

**Authors:** Congwu Du, Kicheon Park, Craig P. Allen, Xiu-Ti Hu, Nora D. Volkow, Yingtain Pan

**Affiliations:** 1 Department of Biomedical Engineering, Stony Brook University, Stony Brook, NY 11794 USA; 2 Department of Microbial Pathogens & Immunity, Rush University Medical Center, Chicago, IL 60612 USA; 3 National Institute on Drug Abuse, Bethesda, MD 20852 USA

**Keywords:** Addiction, Molecular neuroscience

## Abstract

Cocaine profoundly affects both cerebral blood vessels and neuronal activity in the brain. The vasoconstrictive effects of cocaine, concurrently with its effects on neuronal [Ca^2+^]_i_ accumulation are likely to jeopardize neuronal tissue that in the prefrontal cortex (PFC) could contribute to impaired self-regulation and compulsive cocaine consumption. Here we used optical imaging to study the cerebrovascular and neuronal effects of acute cocaine (1 mg/kg i.v.) and to examine whether selective blockade of L-type Ca^2+^ channels by Nifedipine (NIF) (0.5 mg/kg i.v.) would alleviate cocaine’s effects on hemodynamics (measured with cerebral blood volume, HbT), oxygenation (measured with oxygenated hemoglobin, HbO_2_) and neuronal [Ca^2+^]_i_, which were concomitantly measured in the PFC of naive rats. Our results show that in the PFC acute cocaine significantly reduced flow delivery (HbT), increased neuronal [Ca^2+^]_i_ accumulation and profoundly reduced tissue oxygenation (HbO_2_) and these effects were significantly attenuated by NIF pretreatment. They also show that cocaine-induced vasoconstriction is distinct from its increase of neuronal [Ca^2+^]_i_ accumulation though both of them contribute to hypoxemia and both effects were attenuated by NIF. These results provide evidence that blockade of voltage-gated L-type Ca^2+^ channels might be beneficial in preventing vasoconstriction and neurotoxic effects of cocaine and give support for further clinical investigations to determine their value in reducing cocaine’s neurotoxicity in cocaine use disorders.

## Introduction

Cocaine is not only a highly addictive drug but also one associated with significant neurotoxicity, including cerebral strokes, transient ischemic attacks as well as seizures [1–4]. Clinical studies using brain imaging have documented profound reductions in cerebral blood flow (CBF) in the brain of cocaine users [5, 6], and preclinical optical imaging studies have reported similar findings in rodent models of cocaine exposure [7–9]. However, the mechanisms associated with cocaine-induced CBF reductions and ischemia are not well understood, but may result from direct vasoconstriction elicited by cocaine-induced [Ca^2+^]_i_ increases in vascular smooth muscle cells [10] and/or by parallel increases in neuronal activity that exacerbate tissue hypoxemia [11]. Indeed cocaine alters neuronal ionic mechanisms [12], including neuronal K^+^ and Ca^2+^ conductance and influx/efflux [13]. In pyramidal neurons from the medial prefrontal cortex (PFC) cocaine increased voltage-sensitive calcium currents in response to membrane depolarization. Previously, we showed that in rodents chronically exposed to cocaine, an acute dose of cocaine triggered a long lasting reduction in CBF and persistent increases in deoxygenated hemoglobin ([HbR]) and in intracellular calcium concentrations ([Ca^2+^]_i_), which we measured in the somatosensory cortex and that were associated with behavioral manifestations of temporal paralysis [14, 15]. Acute cocaine also triggered vasoconstriction and ischemia in naive rats but these effects were shorter lasting than in the chronically exposed rats suggesting sensitization with repeated exposure. Thus cocaine’s vascular effects and its ability to increase neuronal [Ca^2+^]_i_ is likely to contribute to its neurotoxicity.

The elevated intracellular [Ca^2+^]_i_ accumulation resulting from cocaine exposures is associated with its actions on L-type Ca^2+^ channels (L-channels) [16]. Studies have shown that Ca^2+^ antagonists can modify cocaine-induced behavioral effects including motor activity and reinforcement [8, 17]. Acute cocaine abnormally increases Ca^2+^ influx in cortical neurons [18], most likely via L-channels, whereas chronic cocaine up-regulates L-channels in pyramidal cortical neurons [13]. Some have suggested that L-type Ca^2+^-channel blockers (CCBs) such as nimodipine, which prevented cocaine-induced motor stimulation, might reduce cocaine’s rewarding effects [19]. In vitro studies in the medial PFC showed that L-channel blockade abolished chronic cocaine-induced increases in Ca^2+^ influx through voltage-gated Ca^2+^ channels in pyramidal neurons [13]. Ca^2+^-channel blockers can also reduce negative outcomes from cocaine-induced cerebral ischemia and stroke by buffering cocaine-induced vasoconstriction [20]. Here, we hypothesize that blockade of L-channels would ameliorate cocaine-induced increases in neuronal [Ca^2+^]_i_ and vasoconstriction. To test this, we used our multi-modality optical image technique to study the cerebrovascular and neuronal effects of acute cocaine in the PFC and examined the efficacy of the L-type Ca^2+^-channel blocker, Nifedipine (NIF) in alleviating cocaine’s effects on hemodynamics (measured by HbT and oxygenated hemoglobin, HbO_2_) and neuronal [Ca^2+^]_i_ in vivo. We studied the PFC since this brain region is critical for executive function and its disruption is implicated in the impairments in self-regulation and compulsive drug consumption in cocaine addiction.

## Material and method

### Animals and experimental design

All experimental procedures were approved by the Institutional Animal Care and Use Committee at Stony Brook University. F344 adult male rats (*n* = 17) were used; female rats were not included to avoid the confounds from the effects of sex hormones on cerebral blood vessels [21] and on neuronal excitability in response to cocaine [22]. Animals were randomly assigned into four experimental groups as detailed in Table 1. Experimenters were unblind to the conditions as drugs needed to be prepared fresh to prevent dissolution. Rats were single-housed under a 12:12 h light/dark cycle, with ad libitum access to food and water.

**Table 1:** Animal groups and experimental design.

Animal group/experiment	Intravenous drug challenge	Detection/imaging
Group 1. Determine dose of Nifedipine (*n* = 3)	Nifedipine (0.1 mg/kg per 10 min)	[Ca^2+^]_i_ fluorescence
Group 2. Effects of Nifedipine on physiology (*n* = 5)	Nifedipine (0.5 mg/kg)	Blood pressure (MABP), [Ca^2+^]_i_ fluorescence, [HbT]
Group 3. PFC responses to cocaine with vehicles (*n* = 4)	(a) First: pretreatment with vehicle followed by cocaine (1 mg/kg) 30 min later	[HbO_2_], [HbR], [HbT]
	(b) Second: and 2 h later, pretreatment with vehicle followed by cocaine (1 mg/kg) 30 min later	
Group 4. PFC responses to cocaine with vehicle or Nifedipine (*n* = 5)	(a) First: pretreatment with vehicle followed by cocaine (1 mg/kg) 30 min later	[Ca^2+^]_i_ fluorescence, [HbT], [HbO_2_]
	(b) Second: and 2 h later, pretreatment with Nifedipine (0.5 mg/kg) followed by cocaine (1 mg/kg) 30 min later	

### Virus infusion to express GCaMP6f in neurons in PFC

To measure changes in intracellular calcium ([Ca^2+^]_i_) in neurons, we used a genetically encoded calcium indicator, AAV1.Syn.GCaMP6f.WPRE.SV40 virus (Penn Vector Core). Animals were anesthetized with isofluorane, mounted on a stereotaxic frame, and their scalps opened. The GCaMP6f virus was then infused into the right PFC (A/P: +3; M/L: 0.8); two infusions of 0.5 μl were made (D/V: −1.4 and −1 mm from skull) at a rate of 0.2 μl/min, and the injector was left in place for 20 min following each infusion to allow for diffusion and then the scalp was closed. At the time of imaging, 3–4 weeks had elapsed since virus infusion to allow for GCaMP6f expression.

### Surgical preparation

At 3–4 weeks after viral injection, an optical window was implanted over the PFC for imaging [23]. The rats were anesthetized with isofluorane, and a 16-gauge IV catheter was inserted into their trachea (Angiocath, BD) and attached to a respirator to control breathing. An incision was made proximal to the left hind limb to expose the left femoral artery and vein, and catheters (0.58 mm ID, 0.99 mm OD, Scientific Commodities Inc.) were inserted into the femoral artery to monitor arterial blood pressure, and into the femoral vein for drug delivery during imaging. Rats were mounted on a stereotaxic frame (Kopf 900) and a 4 × 6 mm^2^ portion of skull was removed above the frontal cortex (A/P: +1 to +5; M/L: −3 to +3). The dura was carefully removed to expose the brain surface and covered with 1.25% agarose gel and a cover glass (0.15 mm thick; VWR micro-cover glass) affixed to the skull using crazy glue (Gorilla Glue). The animal’s mean arterial blood pressure (MABP; mean = 82.28, SE = 1.88), body temperature (~37–38 °C) and respiration (~40–45 breaths/minute) were monitored and recorded (Small Animal Monitoring and Gating system, model 1025 L, SA Instruments Inc.).

### Drugs and preparation

Cocaine-HCl was provided by the NIDA drug supply program and Nifedipine was purchased from MilloporeSigma (N-7634). Drug solutions were freshly prepared before imaging. Cocaine was dissolved in 0.9% saline solution. Nifedipine (NIF) was dissolve in DMSO (e.g., 50 mg/ml) and mixed with saline (0.5 mg/kg NIF administered in 0.25 ml i.v.). For the vehicle (VEH) we used 5% DMSO in saline (0.25 ml administered i.v.).

### Optical imaging over PFC

For in vivo imaging of the PFC (A/P: +1 to +5; M/L: −3 to +3), we used a multi-modality image platform (MIP) developed in our laboratory using procedures previously described [11, 14, 24]. As illustrated in Fig. S1 (Supplementary Materials), the cranial window was sequentially illuminated by three LEDs (λ_1_ = 568 nm, λ_2_ = 630 nm and λ_excitation_ = 488 nm; Spectra Light Engine, Lumicor), at a rate of 12.5 Hz/channel. Images were captured with MIP image probe connected to a sCMOS camera (pixel size: 6.5 μm; Zyla 4.3, Andor) using modified Solis software (version 4.26, Andor). The MIP allowed us to simultaneously image changes in Ca^2+^ fluorescence and in oxygenated- and deoxygenated-hemoglobin concentrations used to determine changes in total hemoglobin concentration (ΔHbT). Animals were excluded if the cortex was damaged during implantation, or GCaMP6f was insufficiently expressed to allow for accurate imaging.

We measured Ca^2+^ fluorescence and hemodynamics (ΔHbT) responses to cocaine after VEH and after Nifedipine (NIF) pretreatments. Animals in Group 3 and Group 4, were scanned twice: first when pretreated with VEH prior to a first cocaine injection, and then when pretreated with VEH (Group 3) or NIF (Group 4) prior to a second cocaine injection, with approximately 2 h between each cocaine infusion. Each scan consisted of a 10 min baseline, 10 min infusion of VEH or of NIF (0.5 mg/kg), then 10 min to establish a new baseline, followed by a 1 min cocaine infusion (1 mg/kg, 0.1 mL) after which imaging continued for 60 min post cocaine administration.

### Image processing

The hemodynamic changes in the cortical tissue, i.e., the changes in oxygenated hemoglobin (ΔHbO_2_) and deoxygenated hemoglobin (ΔHbR), were calculated from λ_1_ and λ_2_ images obtained from MPI system according to the equation

1 $$\left[ {\begin{array}{*{20}{c}} {{\Delta} {\mathrm{HbO}}_2} \\ {{\Delta} {\mathrm{HbR}}} \end{array}} \right] = \left[ {\begin{array}{*{20}{c}} {\varepsilon _{{\mathrm{HbO}}_2}^{\lambda _1}} & {\varepsilon _{{\mathrm{HbR}}}^{\lambda _1}} \\ {\varepsilon _{{\mathrm{HbO}}_2}^{\lambda _2}} & {\varepsilon _{{\mathrm{HbR}}}^{\lambda _2}} \end{array}} \right]^{ - 1}\left[ {\begin{array}{*{20}{c}} {\ln \left( {R_{\lambda_1}\left( 0 \right)/R_{\lambda _1}\left( t \right)} \right)/L_{\lambda_1}\left( t \right)} \\ {\ln \left( {R_{\lambda_2}\left( 0 \right)/R_{\lambda_2}\left( t \right)} \right)/L_{\lambda _2}\left( t \right)} \end{array}} \right]$$

where ε^λ1^_HbO2_, ε^λ1^_HbR_, ε^λ2^_HbO2_, and ε^λ2^_HbR_ are the molar extinction coefficients for HbO_2_ and HbR at the two wavelengths, R_λ1_(*t*) and R_λ2_ (*t*) are the measured diffuse reflectance matrices (2-D images) at these wavelengths and L_λ1_ (*t*) and L_λ2_ (*t*) are the path lengths of light propagation [25, 26]. The sum of the changes in oxygenated hemoglobin (ΔHbO_2_) and deoxygenated hemoglobin (ΔHbR) produces the changes in the total hemoglobin (ΔHbT), which can be so-called as the changes in the cerebral blood volume within the cortex [24].

Changes in intracellular ([Ca^2+^]_i_) were calculated from fluorescence (λ_excitation_ = 488 nm, λ_emission_ ≧ 510 nm). To minimize the effect of absorption changes within the tissue (due to blood flow changes) on the calcium fluorescence measurement, the fluorescence intensity in regions expressing GCaMP were normalized to the background intensity detected from the equivalent left hemisphere region, which had no expression (e.g., left side of PFC in the top panel of Fig. S1C). Results are presented as percent change from baseline to control for differences in GCaMP expression between animals.

**Fig. 1:**
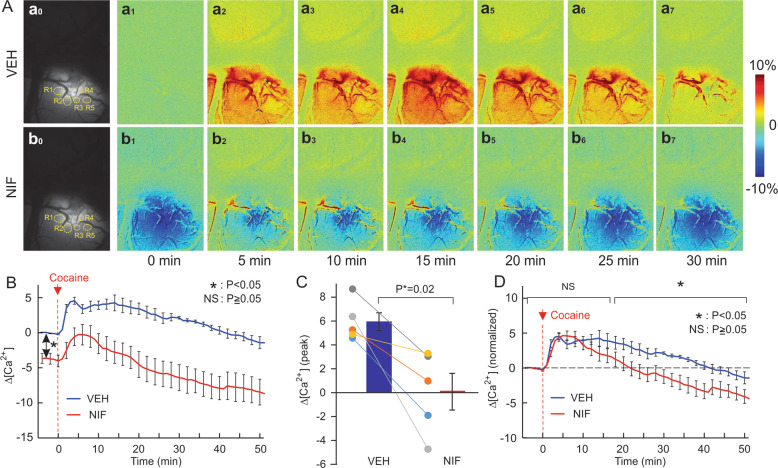
Prefrontal neuronal [Ca^2+^]_i_ fluorescence in response to cocaine challenge with or without NIF pretreatment. **A** Representative [Ca^2+^]_i_ fluorescence images showing the spatial and dynamic changes in response to cocaine (1 mg/kg, i.v.) in PFCs after vehicle pretreatment (VEH, upper panel) and NIF-pretreatment (NIF, lower panel); It shows that NIF lowered baseline levels of Ca^2+^ activity even after cocaine. **B** Averaged dynamic changes for cocaine-induced [Ca^2+^]_i_ fluorescence without (VEH) and with NIF pretreatment (0.5 mg/kg, i.v., NIF). **C** Comparison of mean peak changes in neuronal [Ca^2+^]_i_ fluorescence (i.e., Δ[Ca^2+^]_i_) triggered by cocaine between vehicle and NIF pretreatment alongside the individual values for each animal (*n* = 5), showing the significant attenuation by NIF. **D** Averaged normalized dynamic change in [Ca^2+^]_i_ following cocaine with vehicle and NIF pretreatment at time *t* = 0 min. Values in graphs correspond to mean and standard errors. ROIs: the regions of interest in the tissue that did not have visible blood vessels to minimize the light attenuation from blood passing through vessels. For each animal, five ROIs were selected in the GCaMP6f-expressed regions. ^*^p < 0.05; NS: not significant.

**Fig. 2:**
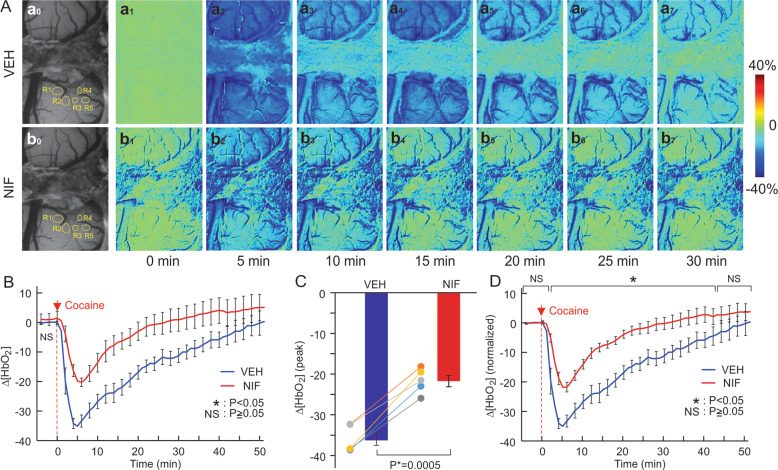
NIF decreased cocaine’s hypoxemic effects in PFC. **A** Representative images sowing dynamic changes in response to cocaine (1 mg/kg, i.v.) after vehicle pretreatment (VEH, upper panel) and after NIF-pretreatment (NIF, lower panel). **B** NIF pretreatment (0.5 mg/kg, i.v.) did not affect baseline [HbO_2_] but significantly attenuated cocaine-induced [HbO_2_] reductions. **C** Comparison of cocaine-induced peak decreases in tissue oxygenation (ΔHbO_2_) between vehicle and NIF pretreatment alongside the individual values (*n* = 5). **D** Normalized [HbO_2_] values (to baseline VEH) for baseline (*t* = 0) and after cocaine showing the attenuation of [HbO_2_] reductions with NIF pretreatment. Values in graphs correspond to mean and standard errors. ROIs: the regions of interest. For a given animal these ROIs were placed in the same location in hemodynamic channels to the Ca^2+^ fluorescence channel. ^*^p < 0.05; NS: not significant.

**Fig. 3:**
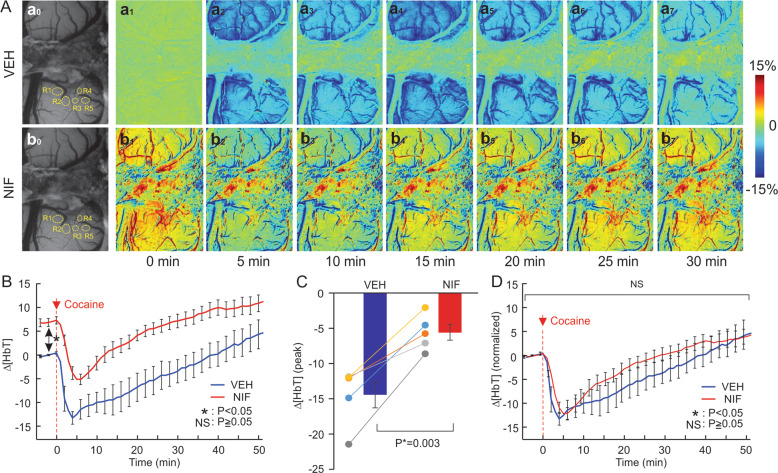
Pretreatment of NIF increased blood volume and attenuated its absolute but not its relative reduction. **A** Representative images of the dynamic changes in cerebral blood volume (∆[HbT]) in response to cocaine (1 mg/kg, i.v.) in PFCs after vehicle (VEH, upper panel) and NIF-pretreatment (NIF, lower panel); Panels Aa_0_ and Ab_0_ show representative images for baseline before and after NIF (0.5 mg/kg, i.v.), respectively. **B** Dynamic measures of cocaine-induced cerebral blood volume (∆[HbT]) changes with vehicle and with NIF pretreatment (0.5 mg/kg, i.v.). **C** Comparison of the integrated decreases in ΔHbT (*n* = 5) between vehicle and NIF pretreatment after cocaine. **D** Normalized dynamic change in (∆[HbT]) following cocaine at time *t* = 0 min. These results show that NIF pretreatment (0.5 mg/kg, i.v.) dilates vessels, elevating baseline cerebral blood volume and attenuated the non-normalized cocaine-induced (∆[HbT]) decreases. Values in graphs correspond to mean and standard errors. ROIs: the regions of interest. For a given animal these ROIs were placed in the same location in hemodynamic channels to the Ca^2+^ fluorescence channel. ^*^p < 0.05; NS: not significant.

To quantify cocaine-induced changes in neuronal [Ca^2+^]_i_ fluorescence and hemodynamics (HbO_2_ and HbT) and to compare differences between VEH and NIF pretreatment, we selected regions of interest (ROIs) within the PFC where there were no visible blood vessels to minimize the light attenuation from high volume of blood passing through vessels. For each animal we extracted five ROIs in the GCaMP6f-expressed region (as demonstrated Fig. 1a_0_) and used the same ROI location for the hemodynamic channels (HbO_2_ in Fig. 2a_0_, HbT in Fig. 3a_0_) to ensure these multiple parameters were measured in the same region.

### Statistical analysis

Results are reported as mean ± standard error (SE). Data was analyzed using two-way repeated measure ANOVAs, and Bonferroni corrected t-tests were used for post hoc analyses (SigmaStat, Systat Software, Inc.). Statistical tests were performed using SigmaStat software (Systat Software Inc.), and alpha levels were set at 0.05, with only significant effects reported.

## Results

### Optimization of Nifedipine (NIF) administration in vivo

To determine what doses of NIF would block intracellular Ca^2+^ in neurons while minimally affecting blood pressure we administered 0.1 mg/kg NIF every 10 min (shaded time periods in Fig. S2A, Supplementary Materials), while monitoring the changes in [Ca^2+^]_i_ fluorescence. After the 5th infusion (i.e., 0.5 mg/kg NIF in total), the [Ca^2+^]_i_ fluorescence signal did not change further for more than 10 min, indicative of stabilization, so this dose (0.5 mg/kg, i.v.) was selected for pretreatment before the cocaine challenge.

Figure S2B (Supplementary Materials) summarizes the changes in MABP as a function of time. After NIF (0.5 mg/kg, 0.25 ml i.v.) infusion (shaded time period in Fig. S2B), MABP decreased from 85.3 ± 1.45 mmHg at baseline to 66.3 ± 2.96 mmHg within 5–6 min of infusion and gradually recovered to 76.0 ± 2.08 mmHg at *t* = 12 min remaining stable thereafter for the time period of recording. Although 0.5 mg/kg NIF reduced MABP, it did not decrease it beyond the range for autoregulation [27], and prior to cocaine infusion it had stabilized at a level that did not differ from baseline (*p* = 0.43).

### Nifedipine (NIF) decreased neuronal [Ca^2+^]_i_ and increased blood volume in PFC

We hypothesize that NIF would diminish cocaine-induced [Ca^2+^]_i_ increases in neurons and dilate vessels increasing blood supply to PFC. Figure S3 (Supplementary Materials) shows time courses of [Ca^2+^]_i_ fluorescence and changes in cerebral blood volume (measured by changes in HbT) induced by NIF (0.5 mg/kg, i.v., over 10 min) following vehicle infusion. After 2–3 min of NIF infusion, mean neuronal [Ca^2+^]_i_ gradually decreased, with a peak reduction of 3.66 ± 0.74% occurring after 15 min of NIF induction (i.e., at *t* = 20 min in Fig. S3)_,_ and plateauing thereafter. The decrease in [Ca^2+^]_i_ with NIF differed significantly from baseline (*p* < 0.001, Fig. S3C). Meanwhile, significant increases in cerebral blood volume (ΔHbT) were observed following NIF that stabilized at 9.2 ± 1.65% over the baseline after 15 min of induction (i.e., at *t* = 22 min, *p* < 0.001, Fig. S3B). These results indicate that NIF concurrently triggered vascular and neuronal [Ca^2+^]_i_ changes.

### Nifedipine (NIF) reduced the duration of cocaine-induced changes in cortical activity

To compare cocaine’s effects on PFC with and without blockade of L-type Ca^2+^ channels by NIF each animal was administered cocaine twice, i.e., first with vehicle and then with NIF as pretreatment before cocaine administrations. To minimize lingering effects from the first cocaine dose we gave the second cocaine infusion 2 h after the first one. Figure S4 (Supplemental material) compares the hemodynamic responses (e.g., [HbO_2_], [HbR], and [HbT]) to cocaine (*n* = 4) between the first and the second cocaine challenge when given after vehicle (5% DMSO in saline) pretreatments, which indicates that, when rats were pretreated with vehicle twice, there was no significant difference in the PFC response to cocaine.

Cocaine triggered significant changes in neuronal [Ca^2+^]_i_ in PFC that differed when given after vehicle or after NIF pretreatment (Fig. 1). To obtain these measures we obtained a 10 min baseline scan, followed by a 10 min infusion of vehicle (5% DMSO in saline) or NIF (0.5 mg/kg i.v., 0.25 ml) scan, then a 10 min scan to establish a new baseline, followed by a 1 min cocaine infusion (1 mg/kg i.v., 0.1 mL) with imaging continuing for 60 min post cocaine administration. Figure 1Aa_0_, Ab_0_ shows representative images of GCaMP6f neuronal expression before and after NIF infusion, respectively. Figure 1Aa_1_–a_7_, Ab_1_–b_7_ shows the dynamic changes in GCaMP6f [Ca^2+^]_i_ fluorescence in response to cocaine for VEH and NIF pretreatment, respectively. Figure 1Aa_1_–a_7_ shows that with VEH pretreatment cocaine increased neuronal Ca^2+^ fluorescence. Two-way repeated ANOVA showed a significant treatment and time effect [*F*(64, 256) = 2.99, *p* < 0.001] (*n* = 5, ROIs = 5/animal). Post hoc comparisons showed that in the VEH pretreated group [Ca^2+^]_i_ fluorescence increased from 2 to 24 min (*p* < 0.05) and returned to baseline after 25 min (*p* > 0.05). In the NIF pretreated group, [Ca^2+^]_i_ fluorescence increased from 3 to 12 min (*p* < 0.05) and returned to baseline after 13 min (*p* > 0.05). Figure 1B shows that NIF by itself reduced intracellular Ca^2+^ concentration, with an average of −3.70 ± 0.7% decrease from baseline (*p* < 0.01). Figure 1Ab_2_–b_7_ shows that even after cocaine, Ca^2+^ fluorescence with NIF remained lower (Fig. 1A and red curves in Fig. 1B,*)* than the vehicle baseline. The peak neuronal [Ca^2+^]_i_ fluorescence increase induced by cocaine corresponded to 5.94 ± 0.7% for VEH and to 0.10 ± 1.5% for NIF pretreatment (*p* = 0.02) (Fig. 1C).

After normalizing the NIF baseline (NIF) to levels equivalent to the vehicle baseline (VEH *t* = 0) (Fig. 1D), one can observe that cocaine induced a short lasting increase in Ca^2+^ that by 25 min was lower than the baseline (Fig. 1D, [*F*(64, 256) = 10.31, *p* < 0.001]). NIF did not alter the peak amplitude of cocaine-induced increase in Ca^2+^ (4.0 ± 0.83%) but shortened its duration_._ A two-way repeated ANOVA on [Ca^2+^]_i_ changes showed a significant drug and time interaction effect ([*F*(64, 256) = 2.99, *p* < 0.001]). Comparisons of [Ca^2+^]_i_ changes for the same time points for the VEH and NIF pretreatment are shown in Fig. 1D and significant differences are summarized in Table S1 (Supplementary Materials). Analyses showed that, the normalized [Ca^2+^]_i_ responses to cocaine did not differ between VEH and NIF during the time period of *t* = −4–17 min (*p* = 0.97~0.06) but the [Ca^2+^]_i_ changes recovered much faster with NIF than with VEH, thus showing significant differences during *t* = 18–60 min (i.e., *p* = 0.04~0.004, Table S1A).

### Nifedipine (NIF) did not change basal oxygenation but attenuated cocaine-induced [HbO_2_] reduction

Acute cocaine decreased [HbO_2_] after vehicle and NIF pretreatment attenuated this decrease and its duration (Fig. 2). Panels Aa1–a7 and Ab1–b7 (Fig. 2) show representative images of percent change in [HbO_2_] concentration over time following cocaine injection at *t* = 0 min with VEH and NIF pretreatment, respectively. Two-way ANOVA on the comparisons of Δ[HbO_2_] of VEH and NIF showed a significant interaction between pretreatment and time ([*F*(64, 256) = 3.07, *p* < 0.001]). For VEH, cocaine induced a persistent decrease that returned to baseline at 30 min (*t* = 1~32 min; *p* < 0.001, *t* = 33 min; *p* = 0.15) (Fig. 2B), whereas with NIF pretreatment, cocaine’s effect was reduced in amplitude by 13.21 ± 3.3%, (*p* < 0.001) and duration (<15 min) (*p* = 0.01) (Fig. 2B). NIF pretreatment did not cause a significant change in baseline [HbO_2_] (*p* = 0.17) (Fig. 2B). The peak decreases in [HbO_2_] by cocaine with VEH corresponded to 36.0 ± 1.5% and with NIF to 21.6 ± 1.4% (*p* < 0.05) (Fig. 2C). Figure 2D shows the normalized time courses of [HbO_2_] changes in response to cocaine with VEH or NIF pretreatment. Following VEH cocaine reduced [HbO_2_], which remained below baseline at 45 min post cocaine (*p* = 0.03), whereas after NIF the recovery was significantly faster (*t* = 31 ± 7.5 min post cocaine) (*p* = 0.008). The comparison of VEH and NIF responses to cocaine for the equivalent time points showed no differences in HbO_2_ changes from *t* = −4–1 min (*p* = 0.97~0.45, Supplementary Table S1B) and significant differences between *t* = 2 and 43 min (*p* < 0.001~*p* = 0.04,) followed by recovery to baseline with no significant differences from *t* = 44~60 min (*p* = 0.08~0.23, Table S1B).

### Nifedipine (NIF) mitigates cocaine’s reduction of blood volume in PFC

Acute cocaine reduced cerebral blood volume as measured by changes in HbT (Fig. 3). Figure 3A (Aa_1_–a_7_) shows representative images for percent change in HbT (∆[HbT]) over time following cocaine injection from *t* = 0 min, illustrating the ∆[HbT] decreases. Figure 3A_a1_, A_b1_ shows ∆[HbT] images before and after NIF showing the increase in [HbT] after NIF (average increase of 6.9 ± 0.83% *p* = 0.003). Two-way repeated ANOVA for the comparisons between Veh-Coc and NIF-Coc showed a significant interaction of Pretreatment and Time ([*F*(64, 256) = 1.48, *p* = 0.02]) but the pretreatment effect was not significant (*p* = 0.39). Cocaine’s reduction of ∆ [HbT] was significantly shortened from 39.4 ± 5.8 min after VEH to 9.8 ± 2.1 min after NIF *p* = 0.004 (Fig. 3B). We also compared cocaine-induced changes (∆[HbT]) integrated over time before they returned to baseline and showed significant differences between VEH and NIF that corresponded to −14.45 ± 1.8% and −5.61 ± 1.1%, respectively (*p* = 0.003). Figure 3D shows the normalized dynamic changes in ∆[HbT] following cocaine at time *t* = 0 min, showing no significant differences between VEH and NIF (*p* > 0.05, Supplementary Table S1C).

### Cocaine-induced hypoxemia was associated with both its reduction of blood volume and its neuronal Ca^2+^ increases, and NIF buffered these changes

**Fig. 4:**
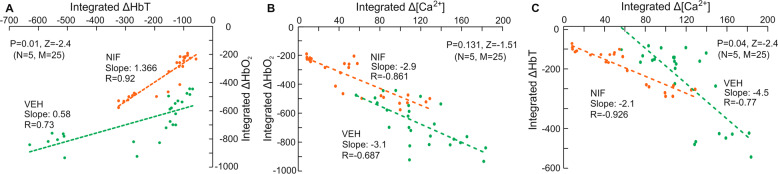
Linear regression analyses for change in ΔHbT, ΔHbO_2_, Δ[Ca^2+^]_i_ signaling induced by cocaine. **A** Linear regression between cocaine-induced ΔHbT and ΔHbO_2_. **B** Linear regression between cocaine-induced Δ[Ca^2+^]_i_ and ΔHbO_2_. **C** Linear regression between cocaine-induced Δ[Ca^2+^]_i_ and ΔHbT. Green markers: with vehicle treatment; Orange markers: with NIF pretreatment.

In order to determine the extent to which the reductions in HbO_2_ with cocaine were due to reductions in blood volume (HbT) or due to increases in oxygen utilization from neuronal activation ([Ca^2+^]_i_) we computed the regression plots between ΔHbO_2_ and ΔHbT (Fig. 4A) and between ΔHbO_2_ and Δ[Ca^2+^]_i_ (Fig. 4B) and compared these associations between VEH and NIF. The regression analyses were preformed across all animals including all ROI and time points (*n* = 5 and multiple ROIs (*m* = 5). The correlations between cocaine-induced changes in HbT and HbO_2_ were significant both for VEH (*r* = 0.73, *p* < 0.001) and for NIF (*r* = 0.92, *p* < 0.001). However, the regression slopes differed significantly (*Z* = −2.4, *p* = 0.01) such that for a given HbT reduction the HbO_2_ levels for VEH were markedly lower than for NIF indicating that an additional factor (neuronal activation) contributed to the reduced HbO_2_ (Fig. 4A). Supporting this, the regression plots showed significant correlation between cocaine-induced increases in [Ca^2+^]_i_ and decreases in HbO_2_ both for VEH (*r* = −0.687, *p* < 0.001) and NIF (*r* = −0.861, *p* < 0.001), and though the slopes did not differ it showed an attenuated [Ca^2+^]_i_ increase with NIF compared to VEH concomitantly with an attenuated reduction of HbO_2_ (Fig. 4B). Finally to assess if NIF prevented cocaine-induced dissociation of neuronal activity ([Ca^2+^]_i_) and blood delivery (HbT), we computed the regression plots between them (Fig. 4C). We showed that for both Δ[Ca^2+^]_i_ increases were associated with ΔHbT decreases, both for vehicle (*r* = −0.77, *p* < 0.001) and NIF (*r* = −0.93, *p* < 0.001). However, their slopes differed (*p* = 0.04), showing a more constrained cocaine-induced reduction in HbT and increase in [Ca^2+^]_i_ for NIF than for VEH indicating that NIF buffered cocaine’s effects both on blood vessels and on neuronal [Ca^2+^]_i_ increases.

## Conclusion and discussion

Cocaine is one of the most addictive drugs and its use, which has expanded markedly in the past decade, is a major contributor of overdose fatalities in the United States [28]. While cocaine’s dopamine-enhancing properties are believed to underlie its rewarding and addictive effects, its vasoconstrictive effects [29] alongside neuronal Ca accumulation [11, 24] and disruption of neurovascular coupling [30] are likely to damage neuronal tissue and in the PFC contribute to impairments in executive function that facilitate compulsive drug taking [8]. In humans, chronic use of cocaine causes damages to the PFC disrupting CBF [5], metabolic activity [31, 32], structure [33, 34], function [35, 36], and neurotransmission [37–39]. In rodents, chronic cocaine exposure was associated with loss of neurons in the PFC [40], perhaps reflecting overactivation-induced neurotoxicity (excessive neuronal Ca^2+^ accumulation) associated with dysfunction of L-type Ca^2+^ channels [13, 41–44] that would be exacerbated by cocaine’s vasoconstriction effects. The increases in [Ca^2+^]_i_ triggered by cocaine would make the tissue more vulnerable to ischemia secondary to decreases in CBF and to cell damage. Thus, we hypothesized that NIF by preventing cocaine-induced vasoconstriction and excitotoxicity from neuronal [Ca^2+^]_i_ accumulation would minimize tissue hypoxemia.

Our results corroborated our hypothesis and showed that in the PFC acute cocaine significantly reduced flow delivery and increased neuronal [Ca^2+^]_i_ accumulation profoundly reducing tissue oxygenation and these effects of cocaine were significantly attenuated by NIF pretreatment. Our results with NIF are consistent with prior studies that showed that the L-type calcium-channel blocker, isradipine reversed cocaine-induced cerebral ischemia and tissue damage in rodents exposed to chronic cocaine [1, 45, 46]. Interestingly, the antivasospastic effects of isradipine were attributed to blockade of dopamine release into cortical neurons that control the cerebral vasculature [17, 47]. Human studies using SPECT imaging reported that isradipine administered 60 min before cocaine (0.33 mg/kg i.v.) prevented both global and regional ischemia in dopamine-rich brain areas [48]. In the heart, clinical studies showed that pretreatment with NIF protected against cocaine’s depression of myocardial function and decrease in coronary blood flow [10, 49, 50] and intracoronary administration of a Ca-channel blocker reversed cocaine-induced microvascular dysfunction [51].

Our findings show that cocaine-induced vasoconstriction is distinct from its increase of neuronal Ca accumulation, and that NIF affected both processes. Specifically, we showed that while cocaine-induced decreases in blood delivery (ΔHbT) were significantly associated with the reduction in oxygenation (ΔHbO_2_) both for VEH and NIF for a given decrease in blood delivery the reductions in oxygenation were smaller for NIF than for VEH indicating that an additional effect of NIF (neuronal Ca accumulation) contributed to the reduced hypoxemia. Indeed, while cocaine-induced increases in [Ca^2+^]_i_ and decreases in oxygenation were significantly correlated both for vehicle and NIF, the [Ca^2+^]_i_ increase with NIF was attenuated concomitantly with an attenuation of hypoxemia. We also showed that NIF reduced cocaine-induced disruption of neurovascular coupling [11] as evidenced by a more constrained reduction in HbT concomitant to the increase in [Ca^2+^]_i_ for NIF than for VEH, thus indicating that NIF buffered cocaine’s effects both on blood vessels and on the neuronal [Ca^2+^]_i_ increases.

We had previously shown using a rodent model of chronic cocaine self-administration that animals that consumed high doses of cocaine showed sensitized responses to cocaine-induced vasoconstriction in PFC and the concomitant hypoxemia was associated with escalation of cocaine intake [8]. In this chronic model of cocaine administration that emulates human cocaine consumption, NIF pretreatment prevented cocaine’s vasoconstriction and neuronal [Ca^2+^]_i_ increases while decreasing cocaine intake and blocking reinstatement [8]. Here we expand these finding to show that NIF prevents cocaine-induced vasoconstriction and neuronal [Ca^2+^]_i_ accumulation, even in naive animals. Also by systematically studying the associations between cocaine’s effects in blood delivery, neuronal [Ca^2+^]_i_ accumulation and hypoxemia in the same animal we are able to document that hypoxemia induced by cocaine is a function of both its vasoactive effects and its increase in neuronal [Ca^2+^]_i_ content, and that NIF benefits likely stem from it preventing both of these effects.

The mechanism underlying the effects of cocaine on vasoconstriction as well as those underlying neuronal [Ca^2+^]_i_ increases are not fully understood. Although the duration of neuronal [Ca^2+^]_i_ response to cocaine with NIF pretreatment was significantly diminished (*t* > 17 min in Fig. 1D), the peak for the normalized neuronal [Ca^2+^]_i_ increase did not differ from that of the VEH (*t* ≤ 17 min, Fig. 1D). Lack of a peak effect suggests that NIF did not interfere with cocaine-induced activation of NMDARs or their phosphorylation, which increases [Ca^2+^]_i_ influx [52–54]. It could also indicate that NIF did not block the initial increase in intracellular Ca^2+^ release from the endoplasmic reticulum [55]. However, since the dose of NIF used (0.5 mg/kg) was relatively low compared to that used by others [56, 57], it is possible that higher doses might have reduced the peak. Moreover, it is likely that combined blockade of NMDARs and L-channels might have suppressed the increased peak.

Cocaine’s vasoconstriction effects are likely to reflect its sympathomimetic effects but its effects on L-type Ca channel’s function in blood vessels are also likely to contribute [58]. The mechanisms responsible for neuronal [Ca^2+^]_i_ accumulation are also likely to involve cocaine’s effects on L-type [Ca^2+^]_i_ channels in neurons, which are disrupted with chronic exposures [42, 44]. Additionally, chronic cocaine triggers neuroadaptations in glutamate neurotransmission [59] that could further worsen cocaine-induced neurotoxicity. Therefore, combined overactivation of L-channels and glutamate signaling with repeated use of cocaine could exacerbate neuronal [Ca^2+^]_i_ dysregulation and excitotoxicity, as well as worsen vasoconstriction mediated by excessive [Ca^2+^]_i_ in blood vessel smooth muscles. How and to what extent dysfunctional NMDA receptors contribute to cocaine-induced neuronal hyperactivity and [Ca^2+^]_i_ dysregulation in PFC requires further investigation.

Our findings have clinical implication for they provide further evidence of the potential of L-type Ca-channel blockers for the treatment of cocaine use disorders. Further preclinical evidence is expanding on the beneficial effects of L-type Ca-channel blockers in preventing cocaine intake and relapse [17, 58, 60, 61]. To the extent that L-type Ca-channel blockers also block cocaine-induced vasoconstriction in the myocardium [62, 63], they could also help prevent cocaine’s lethality.

A limitation for this study is that we only studied male rats. Though we initially selected only males to avoid the confounds of sex hormones on cerebral blood vessels [21] and on neuronal excitability in response to cocaine [22], it is increasingly evident that sex differences in the response to cocaine are clinically relevant [64–71]. Thus future studies should evaluate the effects of NIF on female rats. Another limitation is the small sample size of our study, yet despite this the large and consistent effects of cocaine and its blockade by NIF enabled us to document significant effects. Also, the studies were done under anesthesia, which confounds our findings since we cannot rule out the possibility that some of cocaine’s effects reflect its interaction with the anesthetic agent [72, 73].

In summary, our findings indicate that blockade of L-channels may be beneficial in reducing cocaine-induced hypoxemia, which could help reduce damage to PFC, and potentially other brain regions and thus of therapeutic value in cocaine use disorder.

## Supplementary information

Supplementary Materials.
