# Supplementary material for: Ca2+ channel blockade reduces cocaine’s vasoconstriction and neurotoxicity in the prefrontal cortex
Source: Transl Psychiatry. 2021 Sep 6;11:459. doi: 10.1038/s41398-021-01573-7 (PMC8421405; doi:10.1038/s41398-021-01573-7)
Supplement: Supplementary file 1 — Supplementary Materials. [file 41398_2021_1573_MOESM1_ESM.docx]

**Supplementary Materials**

**Figure S1 Optical imaging of PFC in vivo**


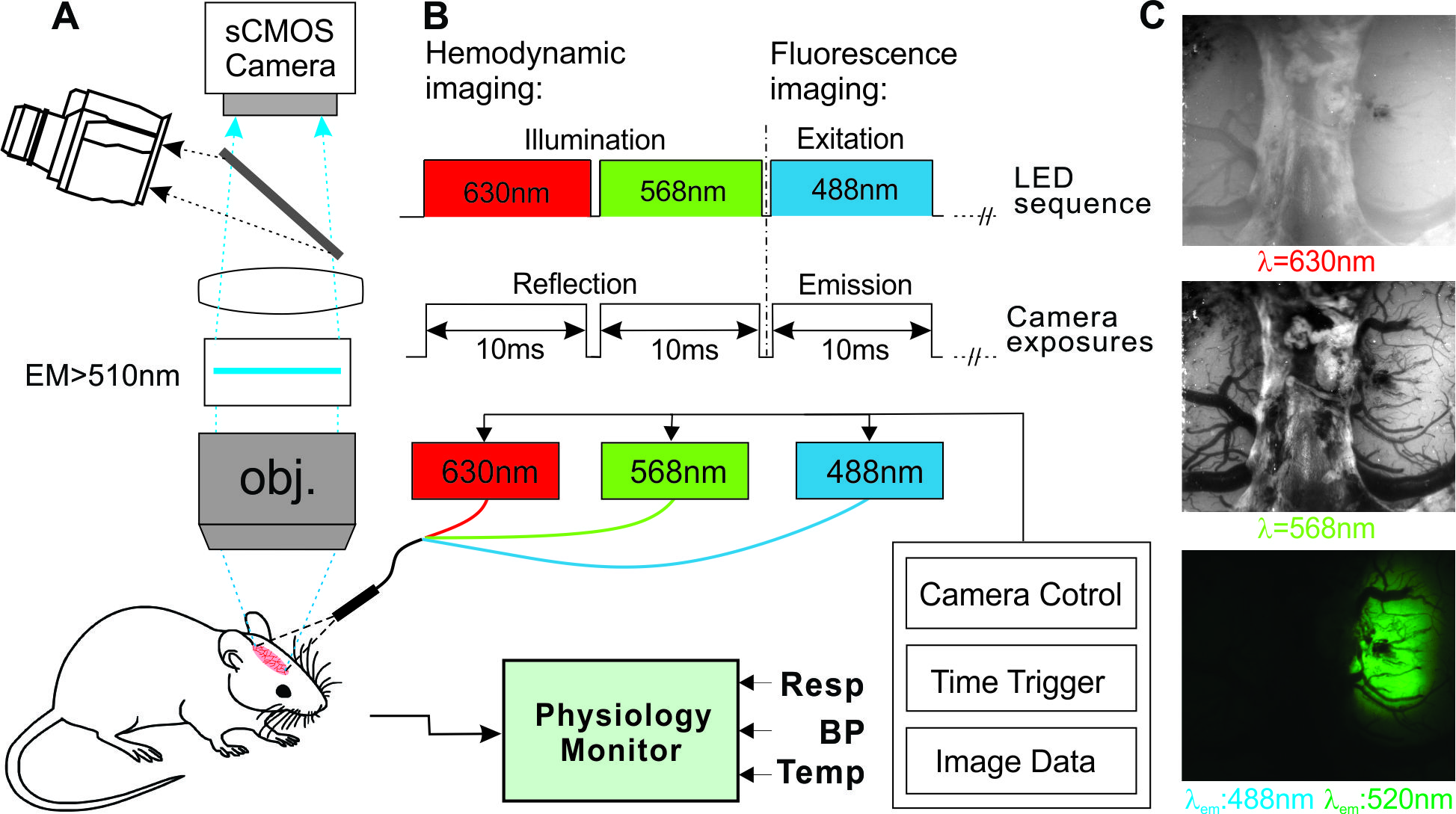


**Figure S1 Schematic diagram to illustrate optical imaging of PFC in vivo**.

**A).** Multimodality imaging platform (MIP) to image neuronal Ca^2+^ fluorescence and the hemodynamic changes in PFC induced by cocaine. **B).** Sequences for simultaneous imaging of fluorescence and hemodynamic changes. **C).** Simultaneous in vivo images of reflection and Ca^2+^ fluorescence from rat prefrontal cortex.

**Figure S2 Optimization of nifedipine (NIF) administration *in vivo***


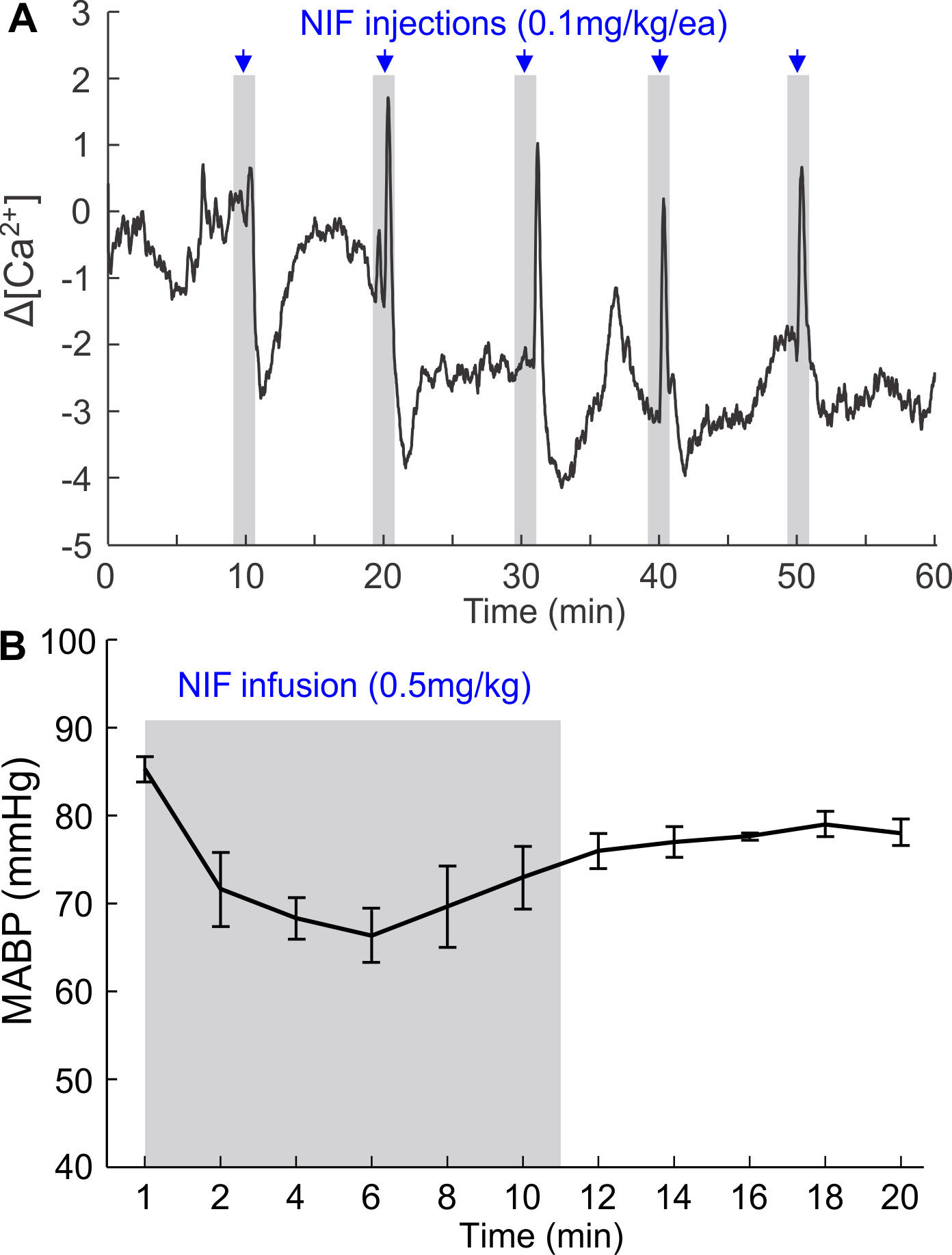


**Figure S2** **Doses of nifedipine (NIF) for in vivo study and analysis of NIF’s effect on physiology.**

**A**): Time course of neuronal Ca fluorescence changes as a function of NIF infusion. 0.1mg/kg NIF was administered every 10min (shaded areas) for n=3 animals; **B**) Effects of NIF on blood pressure (n=5). It indicates that infusion of 0.5mg/kg NIF reduced blood pressure, but it did not decrease it beyond the range for autoregulation. Timelines and doses were chosen to ensure that, prior to cocaine blood pressure was not significantly different from baseline. *Shading* indicates NIF infusion duration. Values in graph correspond to mean and standard errors.

**Figure S3 Nifedipine (NIF) decreased neuronal [Ca^2+^]_i_ and increased blood volume in PFC**


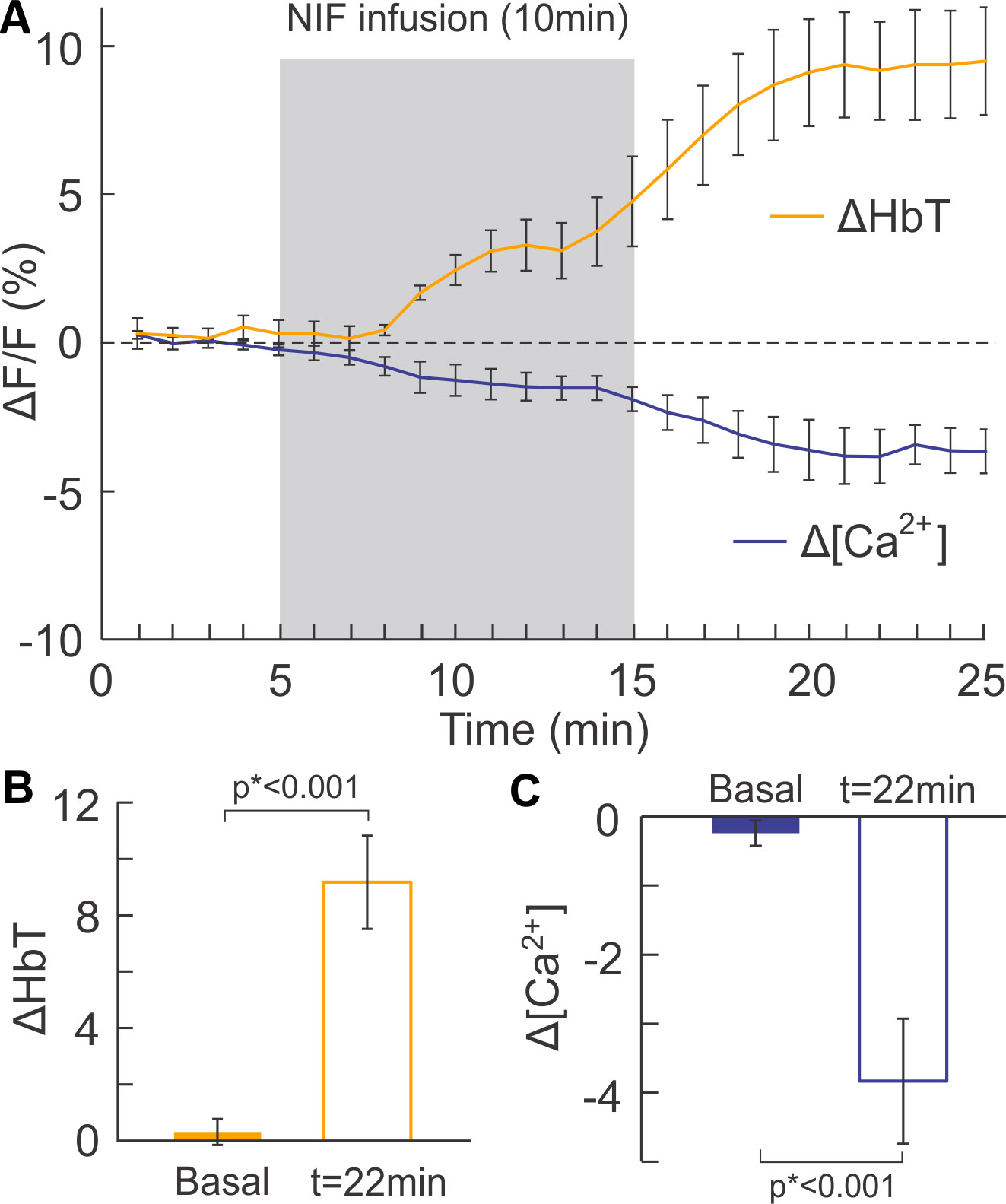


**Figure S3** **Nifedipine reduced intracellular Ca^2+^ and increased blood volume in the PFC.**

**A)** Dynamic changes in Ca^2+^ fluorescence and in hemodynamics induced by infusion of 0.5mg/kg nifedipine (NIF) (n=5). **B)** Comparison of ΔHbT after NIF infusion at t=22 min and at baseline (t=5 min); **C)** Comparison of Δ[Ca^2+^]_i_ after NIF infusion at t=22 min and at baseline (t=5min). Values in graphs correspond to mean and standard errors.

**Figure S4 Hemodynamic response to cocaine in PFC while rats (n=4) were pretreated with vehicle followed an acute cocaine challenge with a 2hrs gap**:

a) pretreatment with vehicle (5% DMSO in saline) followed by cocaine (1mg/kg) at 30min later.

b) 2 hours later, pretreatment with vehicle again followed by cocaine (1mg/kg) at 30 min later.

The experiments showed no significant difference in the hemodynamic responses of PFC to cocaine between the 1st (VEH1) and 2nd (VEH2) cocaine administration.


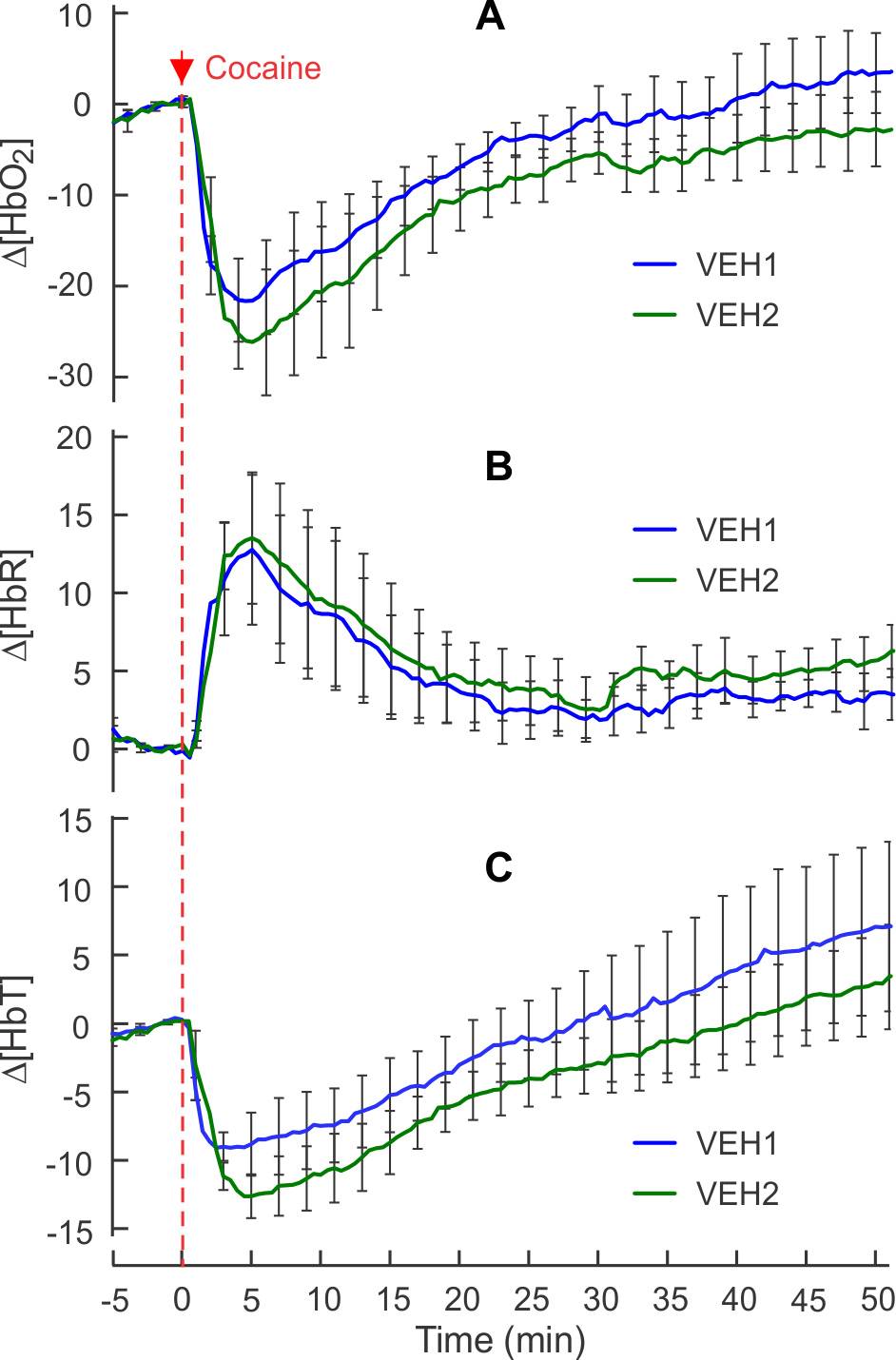


**Figure S4** The cocaine induced hemodynamic changes in Δ[HbO_2_] (**A**), Δ[HbR] (**B**) and Δ[HbT] (**C**) while the animals received the first vehicle with cocaine (blue curves) and the second vehicle (green curves) with cocaine.

**A**. For the 1st cocaine administration, cocaine-induced maximum Δ[HbO_2_] decrease to -21.64% ±4.86% (p<0.001) at was t=4.5min. For the 2nd cocaine administration, the maximal Δ[HbO_2_] decrease to -26.14%±5.5% (p<0.001) was at t=5min. Two-way repeated ANOVA to compare the Δ[HbO_2_] changes between the 1st and the 2nd responses to cocaine in the PFC shows no significant difference (p=0.147).

**B**. For the 1st cocaine administration, cocaine-induced maximum Δ[HbR] increase 12.76%± 4.79% (p<0.001) at was t=5min. For the 2nd cocaine administration, the maximal Δ[HbR] increase to 13.50%±4.20% (p<0.001) was at t=5min. Two-way repeated ANOVA to compare the Δ[HbR] changes between the 1st and the 2nd responses to cocaine in the PFC shows no significant difference (p=0.32).

**C.** For the 1st cocaine administration, cocaine-induced maximum Δ[HbT] decrease to -9.09%± 1.31% (p<0.001) was at t=3.5min. For the 2nd cocaine administration, the maximal Δ[HbT] decrease to -12.64%±1.48% (p<0.001) was at t=4.5min. Two-way repeated ANOVA to compare the Δ[HbT] changes between the 1st and the 2nd responses to cocaine in the PFC shows no significant difference (p=0.371).

**Table S1: p Values of Corresponding Timepoint Comparison of [Ca^2+^ ]_I_, HbO_2_, HbT**

**Responses to Cocaine with Vehicle (VEH) or Nifedipine (NIF) pretreatment**

**A:** Comparison for [Ca^2+^]_i_ responses (p values)

| Time(t) min | -4 | -2 | 0 | 2 | 4 | 6 | 8 | 10 | 12 | 14 | 16 |
| --- | --- | --- | --- | --- | --- | --- | --- | --- | --- | --- | --- |
| **p-value** | **0.97** | **0.95** | **0.95** | **0.21** | **0.86** | **0.27** | **0.60** | **0.59** | **0.59** | **0.06** | **0.06** |
| Time(t) min | 18 | 20 | 22 | 24 | 26 | 28 | 30 | 32 | 34 | 36 | 38 |
| **p-value** | **0.04** | **0.02** | **0.01** | **0.01** | **0.01** | **0.01** | **0.006** | **0.004** | **0.003** | **0.003** | **0.003** |
| Time(t)  min | 40 | 42 | 44 | 46 | 48 | 50 | 52 | 54 | 56 | 58 | 60 |
| **p-value** | **0.003** | **0.02** | **0.02** | **0.02** | **0.03** | **0.02** | **0.01** | **0.01** | **0.008** | **0.01** | **0.004** |

**B:** Comparison for HbO2 responses (p values)

| Time(t)  min | -4 | -2 | 0 | 2 | 4 | 6 | 8 | 10 | 12 | 14 | 16 |
| --- | --- | --- | --- | --- | --- | --- | --- | --- | --- | --- | --- |
| **p-value** | **0.98** | **0.98** | **0.95** | **< 0.01** | **< 0.01** | **< 0.01** | **< 0.01** | **< 0.01** | **< 0.01** | **< 0.01** | **< 0.01** |
| Time(t)  min | 18 | 20 | 22 | 24 | 26 | 28 | 30 | 32 | 34 | 36 | 38 |
| **p-value** | **< 0.01** | **< 0.01** | **< 0.01** | **< 0.01** | **< 0.01** | **< 0.01** | **0.001** | **0.002** | **0.003** | **0.005** | **0.01** |
| Time(t)  min | 40 | 42 | 44 | 46 | 48 | 50 | 52 | 54 | 56 | 58 | 60 |
| **p-value** | **< 0.01** | **0.04** | **0.08** | **0.09** | **0.14** | **0.23** | **0.27** | **0.21** | **0.20** | **0.159** | **0.23** |

**C:** Comparison for HbT responses (p values)

| Time(t)  min | -4 | -2 | 0 | 2 | 4 | 6 | 8 | 10 | 12 | 14 | 16 |
| --- | --- | --- | --- | --- | --- | --- | --- | --- | --- | --- | --- |
| **p-value** | **0.92** | **0.97** | **0.98** | **0.06** | **0.36** | **0.78** | **0.94** | **0.57** | **0.24** | **0.15** | **0.15** |
| Time(t)  min | 18 | 20 | 22 | 24 | 26 | 28 | 30 | 32 | 34 | 36 | 38 |
| **p-value** | **0.16** | **0.13** | **0.12** | **0.13** | **0.18** | **0.15** | **0.16** | **0.19** | **0.22** | **0.24** | **0.31** |
| Time(t)  min | 40 | 42 | 44 | 46 | 48 | 50 | 52 | 54 | 56 | 58 | 60 |
| **p-value** | **0.27** | **0.58** | **0.81** | **0.75** | **0.97** | **0.86** | **0.89** | **0.96** | **0.93** | **0.94** | **0.84** |

Note: Shadow: p< 0.05, indicating significant difference
